# Single‐center experience of LVAD implantation in patients with sickle‐cell trait: A retrospective analysis

**DOI:** 10.1111/jocs.17145

**Published:** 2022-11-22

**Authors:** Khalid Al Khamees, Elena Grasso, Ahmed Ibrahim, Hassane Abdallah, Mohamad I. Adbelhamed, Omer Sayin, Roberto Lorusso

**Affiliations:** ^1^ Department of Adult Cardiac Surgery Prince Sultan Cardiac Center‐Al‐Hassa Hofuf Saudi Arabia; ^2^ Department of Cardio‐Thoracic Surgery Heart & Vascular Centre. Maastricht University Medical Centre (MUMC+) Maastricht The Netherlands; ^3^ Department Research & Biostatistics Prince Sultan Cardiac Center‐Al‐Hassa Hofuf Saudi Arabia; ^4^ Department of Cardiovascular Surgery, Istanbul Medical Faculty Istanbul University Istanbul Turkey; ^5^ Cardiovascular Research Institute Maastricht (CARIM) Maastricht The Netherlands

**Keywords:** hemorrhage, LVAD, sickle cell disease, thrombosis

## Abstract

**Background:**

The most worrisome complications in patients supported by left ventricular assist device (LVAD) are pump thrombosis, embolism, and bleeding. The actual rate of these events in patients with sickle‐cell disease (SCD) has not well investigated. The aim of our study is to evaluate the outcomes of LVAD implantation in patients with sickle‐cell hemoglobinopathy at our institution.

**Methods:**

This retrospective, observational, single‐center study was conducted on patients with sickle‐cell trait (SCT), who underwent LVAD implantation using the HeartMate3 LVAD.

**Results:**

LVAD devices were implanted in four patients with SCT. All procedures were performed successfully. All patients had uneventful post‐implant course. Overall, the mean follow‐up time was 25 months (range 21–28 months) and showed an unremarkable post‐implant course. There was a significant improvement in hematological markers over the follow‐up period.

**Conclusions:**

Despite the limited numbers of patients enrolled in this study, our findings indicate that LVAD surgery is safe in SCD patients and offers remarkable clinical improvement. Further studies are needed to provide more evidence regarding this type of patients undergoing LVAD implantation.

**Abbreviations**
ALT: alanine aminotransferase
ASA: acetylsalicylic acid
AST: aspartate aminotransferase
BMI: body mass index
EF: ejection fraction
EUROMACS: European Registry for Patients with Mechanical Circulatory Support
Hb: hemoglobin
HM3: HeartMate 3
INTERMACS: Interagency Registry for Mechanically Assisted Circulatory Support
LDH: lactate dehydrogenase
LV: left Ventricle
LVAD: left ventricular assist device
LVEDD: left ventricular end‐diastolic dimension
LVESD: left ventricular end‐systolic dimension
MCS: mechanical circulatory support
NYHA: New York Heart Association
PAP: pulmonary artery pressure
SD: standard deviation

## 1 INTRODUCTION

Sickle‐cell disease (SCD) results from a variety of inherited genetic mutations in the hemoglobin‐beta (HBB) gene resulting in defective beta‐globin synthesis and culminating in attenuated oxygen transport.^1^ One particular HBB gene mutation produces an altered beta‐globin molecule known as hemoglobin S (Hb S) with reduced oxygen delivery to end‐organs.

Being regarded as a rare clinical entity, SCD in patients who need open heart surgery at large is poorly addressed in literature.^2^ With diagnostic techniques'advancement, we have encountered many patients with SCD associated with advanced heart failure that necessitates left ventricular assist device (LVAD) implantation. The aim of our present study was to evaluate the outcome of patients with SCD‐related hemoglobinopathy undergoing LVAD implantation at our institution and then followed up after hospital discharge. Hence, we decided to retrospectively look into our data to evaluate the outcomes of LVAD implantation in this type of patients.

## 2 METHODS

At our institution, LVAD implantation program for eligible patients with heart failure was started in year 2017. During the period from 2017 to 2019, the overall number of patients submitted to LVAD implantation using Heartmate3 was 28 patients, and 4 of them (14.3%) had sickle‐cell trait (SCT) hemoglobinopathy. After obtaining approval from the Institutional Review Board, the medical records of the four SCT patients were reviewed and we retrospectively conducted an analysis.

### 2.1 Patient data analysis

Demographics variables, hemodynamic values, and peri‐operative, intra‐operative and postoperative homeostatic parameters and biomarkers are collected and analyzed. Descriptive statistical analysis included categorical data in term of numbers and proportions, while continuous data were analyzed in term of mean ± SD. Analyzed data were presented in tables and graphs. Difference between some essential biomarkers was assessed. Student's *T*‐test and Pearson's chi‐squared test were used to determine whether there were statistically significant differences between the pre, intra and postoperative measured biomarkers. *p* value of <.05 was considered statistically significant and been reported.

### 2.2 Anesthetic management and cardiopulmonary bypass

All patients receive their cardiac medications until the morning of surgery. Oral intake is usually stopped 6 h before LVAD implantation. With glucose check‐up every 2 h and subcutaneous insulin injections as needed, diabetic adult patients receive intravenous fluids according to their body weight, starting at the initiation of preoperative fasting.^2^, ^3^ Sublingual lorazepam (2 mg) is administered 2 h before surgery to decrease anxiety. Anesthesia is induced with either Ketamine iv bolus, Etomidate iv, Fentanyl iv and Esmeron iv. After intubation, all patients were ventilated with 100% oxygen. All invasive procedures are performed while the patients are under deep anesthesia. Arterial blood pressure, central venous pressure, electrocardiogram, saturation with pulse oximetry and rectal temperature are routinely monitored during and after surgery.^4^ Anesthesia is maintained in these cases with Sevoflurane, Propofol iv, Fentanyl iv, and Ketamine iv until the patients are fully awake in the postoperative period. Ultra–fast‐track anesthetic^5^ management is not performed in any of the patients, and all patients were transferred to the intensive care unit while still under full sedation. Patients are extubated when optimal cognitive, hemodynamic, and respiratory functions were achieved. For postoperative pain management, paracetamol is usually administered. Tranexamic acid (50 mg/kg) is used routinely to prevent bleeding complications.^4^, ^5^, ^6^ Perioperative changes in temperature, hemodynamics, and respiratory and metabolic parameters are recorded. Alterations in hemoglobin and hematocrit, blood loss, and transfusion requirement are monitored and documented. Standard hemoglobin electrophoresis is performed to detect the concentrations of HbS and HbA. By local protocol, all patients undergo LVAD implantation on central extra‐corporeal circulation support.

## 3 RESULTS

Base‐line pre‐LVAD implantation patients' characteristics were presented in Table 1.

**Table 1:** Baseline perioperative characteristics

Variable	Patient 1	Patient 2	Patient 3	Patient 4	Mean
BMI kg/m^2^	22.78	29.5	23	29	26.1
Diagnosis	DCM‐postdrug intoxication	DCM‐congenital	DCM	Postpartum cardiomyopathy	NA
EF	12	10	10	12	11.0
INTERMACS profile	3	3	3	3	3
Cardiac Index	1.38	2	2	1.4	1.7
Hb g/dl	13.2	13	9.8	11.9	12.0
Hb A %	59	55.2	63.1	73.3	62.7
Hb A2%	2.7	2.4	2.7	3.2	2.8
HbS %	36.2	41	33.6	24.5	33.8
HbF%	2	1.4	2	0.7	1.5
G6PD deficient	Yes	No	No	No	NA
Platelets 10^3^/μl	225	119	544	306	298.5
Total *Bilirubin* mg/dL	26.5	20.2	34	3.3	21.0
Direct Bilirubin mg/dL	7.7	7.8	16.8	1.6	8.5
Creatinine mg/dL	95	80	88	73	84.0
Urea mg/dL	5.7	2.5	7.3	5.3	5.2
ASA u/l	24	8.5	12.8	11	14.1
ALT u/l	40.2	23	13	12.3	22.1
LDH u/l	220	119	164	133	159.0

Abbreviations: ALT, alanine aminotransferase; AST, aspartate aminotransferase; BMI, body mass index; DC, dilated cardiomyopathy; EF, Ejection fraction; INTERMACS, Interagency Registry for Mechanically Assisted Circulatory Support; Hb, hemoglobin; LDH, lactate dehydrogenase.

Three males and one female were included in the study. The mean age for patients was 33 ± 10.1 years. Overall baseline characteristics of the patients are presented in term as follows: BMI (26.1 ± 3.7), EF:11 ± 1.2, cardiac index was 1.7 ± 0.3, Hb 12 ± 1.6, HbA%62.6 ± 7.8, HbA2% 2.8 ± 0.3, HbS % 34 ± 6, HbF% 2 ± 0.5, Platelets 103/μL 299 ± 181, total Bilirubin mg/dL21 ± 13, direct Bilirubin mg/dl8.5 ± 6, serum creatinine mg/dL 84 ± 10, Urea mg/dL5.2 ± 2, AST u/l 14.1 ± 7, ALT u/l 22.1 ± 13, LDH u/l 159 ± 45. More detailed preoperative characteristics for each patient are shown in Table 1

Two patients underwent aortic clamping and cardiac arrest with warm blood cardioplegia to perform associated procedures. The potential risk of sickling within the coronary arteries with the administration of cold cardioplegia under the cross‐clamp was avoided with an initial normothermic (36°C) blood cardioplegia dose until cardiac arrest followed by a blood cardioplegia dose of 20 ml/kg at normothermic temperature. Subsequently, 10 ml/kg blood cardioplegia was administered every 20 min. Patient 2 underwent combined aortic valve replacement surgery with bioprothesis implantation; the duration of CBP was 188 min and the cross‐clamp was 86 min. Patient 4 underwent combined mitral valve repair; the duration of CBP was 139 min and the cross‐clamp time was 55. The bypass circuit volume was arranged to be 3 times the patient's circulating volume.^7^, ^8^ These volumes were adjusted according to the age, weight, and body surface area of each individual patient to reach a hematocrit value of 30% during CPB in two patients and to decrease HbS levels to ≤10% of circulating hemoglobin. Additional crystalloid, colloid, or red blood cells were added to the CPB circuit as needed according to the desired hematocrit levels.^7^, ^8^ The flow was adjusted as body surface area times cardiac index (2.2–2.4 for the adult patients). To avoid the risk of sickling, rectal temperature was kept around 34°C, while pH was maintained between 7.34 and 7.44.^20^ None of the patient received blood or any blood products transfusion preoperatively. Exchange transfusion was not performed preoperatively and during surgery in all patients. Three patients received all three types of blood components intra‐operatively, while one patient received only platelets and plasma (Table 2). Neither sickling crisis nor acidosis occurred in any patient. There were no complications related to hemoglobinopathy in the immediate postoperative period, and at 3 and 6 months after discharge (no macroscopic or microscopic evidence of hemolysis were seen, nor hematuria or other clinical evidence of sickling). Anticoagulation for LVAD was started with low molecular weight heparin on the ICU after 12–24 h, depending on bleeding amount. No antiplatelet drugs were used in the first few days after LVAD implantation. The following days concomitant warfarin was administered to the patient until INR > 2.0. Target INR for the devices was 2.0–3.0. During postoperative hospital stay, International Normalized Ratio (INR) was daily monitored.^9^ Then, we combined warfarin and acetylsalicylic acid. There was neither mortality nor complication within 30 days, 3 months and 6 months postoperatively. Currently, all patients remain free of significant signs and symptoms of cardiac failure. Patient 1 received heart transplant 22 months after LVAD implantation without complications up to 30 days after heart transplant.

**Table 2:** Intra‐operative findings

Variable	Patient 1	Patient 2	Patient 3	Patient 4
Procedure	LVAD	LVAD + AVR	LVAD	LVAD + MV REPAIR
LVAD type	HeartMate3	HeartMate3	HeartMate3	HeartMate3
CBP/minute	No	188	No	139
Cross‐Clamp	No	86	No	55
Cardioplegia	No	Yes	No	Yes
Blood components transfusion				
PRBC/unit	0	2	2	2
Platelets/unit	2	3	2	1
Plasma/unit	4	5	3	2
Hospital stay(days)	33	40	52	36
Fellow‐up status	Alive	Alive	Alive	Alive

Abbreviations: AVR, aortic valve replacement; CBP, cardiopulmonary bypass; MV REPAIR, mitral valve repair; PRBC, Packed red blood cells.

**Figure 1:**
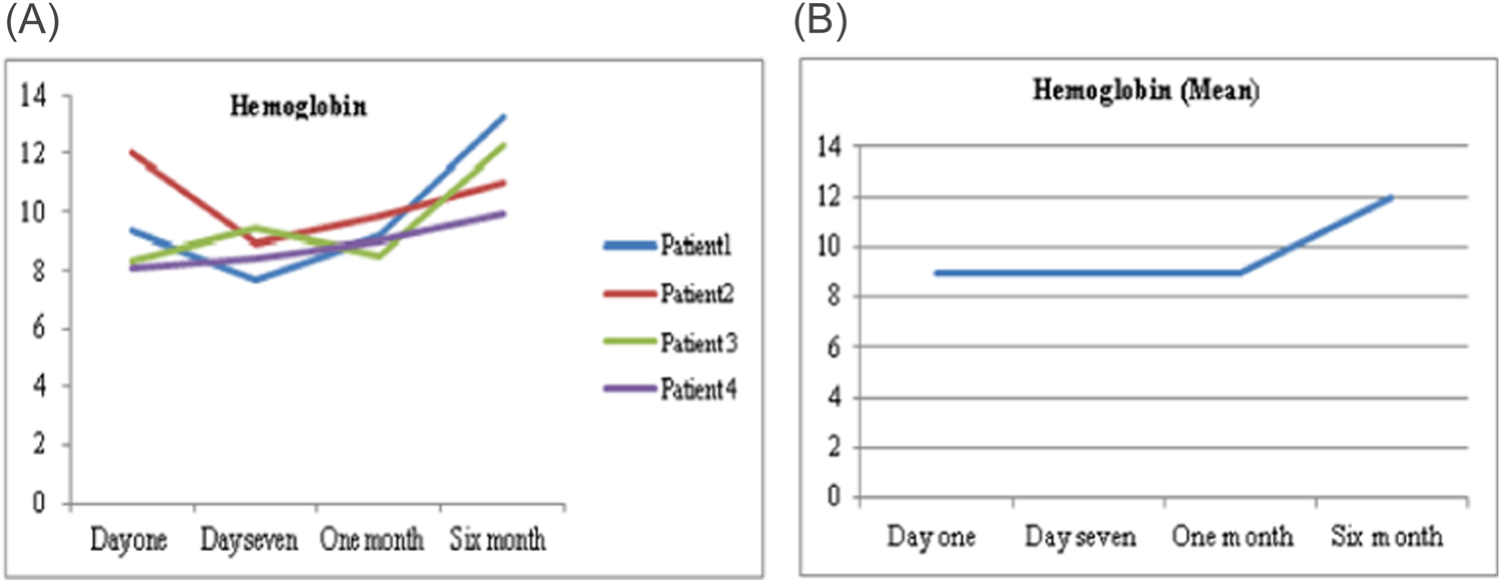
Hemoglobin.

**Figure 2:**
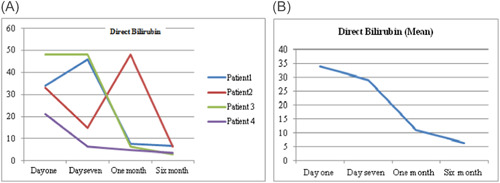
Direct bilirubin.

**Figure 3:**
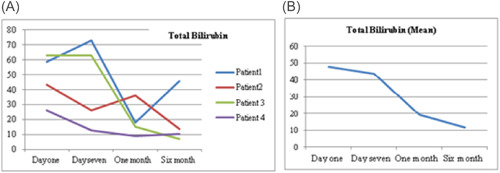
Total bilirubin.

**Figure 4:**
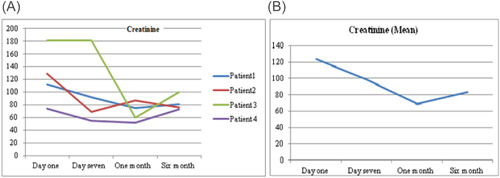
Creatinine.

**Figure 5:**
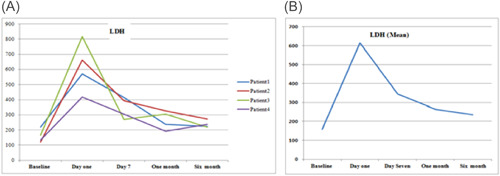
Lactate dehydrogenase.

**Figure 6:**
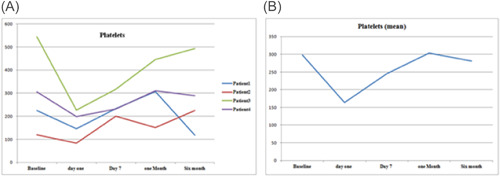
Platelets.

As a routine for our institution the follow‐up of the patients after discharge were performed by VAD Team every week in the outpatient clinic. There were variations in hematological markers among the patients, during follow‐up. Figure 1 shows the mean hemoglobin remained below 10 g/dl after the procedure for all patients until first month of follow‐up, then it gradually increased until it reached 12 g/dl (range 1.3–3.9 g/dl). Mean total bilirubin (Figure 3) decreased from 43.8 mg/dL on the first day of the operation to 11.9 mg/dl after 6‐month of follow‐up (range 8.8–60 mg/dl). While mean direct bilirubin significantly decreased from 34 mg/dL on the first post‐implant day to 6 mg/dl in 6‐month follow‐up respectively (Figure 2). However, mean creatinine (Figure 4) decreased gradually from 124 mg/dl on day one postoperation to 64 mg/dl in first month of follow‐up, then started to increase to 83 mg/dl in the sixth months of follow‐up. The elevation of the mean creatinine level was due to increase in the creatinine level of Patient 3 and Patient 4 (Figure 4). However, there was statistically significant difference in mean creatinine (124 vs. 63 mg/dl; *p* < .001) for Day 1 and after 1‐month of follow‐up respectively (Table 3, Figure 4). There was statistically significant increase in mean of LDH in Day 1 compared to baseline (616 vs.159 U/L; *p* = .002) respectively (Figure 5). However, the level of LDH decreased gradually until it reached 239 U/L in Month 6 of follow‐up. There was a remarkable increase in mean platelet counts for all patients on the first postoperative day compared with baseline mean (164 vs. 299; *p* = .2), but the increase was not statistically significant and was within the normal platelet range. Marked decrease in platelets was observed only inPatient 3 in the first postoperative day. At six‐months follow‐up, the overall mean of platelets count remained within normal range. In spite of decrease on average platelet count at 6 months of follow‐up, we didn't observe any bleeding event in any patient (Figure 6). No patient received platelets transfusion in the postoperative period.

**Table 3:** Postoperative parameters and biomarkers

Variable	Patient 1	Patient 2	Patient 3	Patient 4	Mean
Hospital stay/days	34	25	19	22	25
HeartMate 3 Speed (rpm)	5100	5400	5600	5200	5325
Flow (lpm)	4.4	4.7	4.6	4.3	4.5
Pulsatility Index (PI)	5.7	3.6	2.9	3.8	4.0
Postoperative blood component transfusion/unit					
PRBC unit	6	2	3	2	3.3
Plasma unit	1	0	1	0	0.5
Hemoglobin g/dl					
Day 1	9.3	12	8.3	8.1	9.4
Day 7	7.7	8.9	9.4	8.4	8.6
Month 1	9.2	9.8	8.5	9	9.1
Month 6	13.3	11	12.3	9.9	11.6
Platelets 10^3^/μl					
Day 1	146	84	226	198	163.5
Day 7	233	201	318	231	245.8
Month 1	307	151	446	310	3.03.5
Month 6	118	225	494	289	281.5
Total bilirubin mg/dL					
Day 1	58.8	43.2	63	26	47.8
Day 7	73	26	63	13	43.8
Month 1	18	36	15	9.1	19.5
Month 6	15.7	14	6.9	10.5	11.8
Direct bilirubin mg/dL					
Day 1	34	33	48	21	34.0
Day 7	45.9	14.9	48	6.3	28.8
Month 1	7.7	25.7	6.3	4.7	11.1
Month 6	6.8	12	2.9	3.4	6.3
Creatinine mg/dL					
Day 1	112	129	181	74	124.0
Day 7	92	69	181	55	99.3
Month 1	75	87	60	52	68.5
Month 6	81	76	100	73	82.5
Urea mg/dL					
Day 1	8.5	6	13	6.1	82.5
Day 7	10.8	3.7	7.4	3.2	6.3
Month 1	5.6	3.5	8	3.5	5.2
Month 6	5.6	2.3	5.7	3.3	4.2
AST u/l					
Day 1	138.4	141	46	10	83.9
Day 7	21.1	45	28	22	29.0
Month 1	14.7	27	22	10.7	18.6
Month 6	38	13	11.8	18	20.2
ALT u/l					
Day 1	36.3	28.8	198	15	69.5
Day 7	14.3	49	77	18.9	39.8
Month 1	18.3	58	37.4	9.4	30.8
Month 6	49	12	13.4	26	25.1
LDH u/l					
Day 1	570	661	816	417	616.0
Day 7	416	392	270	305	345.8
Month 1	237	325	304	191	264.3
Month 6	223	273	220	238	238.5

Abbreviations: lpm, liter per minutes; rpm, revolutions per minutes.

## 4 DISCUSSION

Sickle‐cell hemoglobinopathy is a recessively inherited genetic disorder. Approximately 5% of the whole world population carries a potentially pathological SCD‐related gene. SCD is frequently seen among Afro‐Caribbean population but is also found in India, the Middle East, and Southern Europe. The prevalence is even higher in areas endemic for malaria, with SCT reaching around 25% in some parts of Africa and up to 60% in some areas of Saudi Arabia.^1^, ^10^ It results from the mutation of the substitution of adenine for thymidine, which further ends up matching with valine rather than glutamine at the sixth codon of chromosome 11‐globin gene.^11^, ^12^ The condition may present as SCD, the severe form of which is the homozygous genotype (HbSS), in which the fractional concentration of HbS ranges between 70% and 98%. SCD may also manifest itself as SCT, which is rather benign and more common among populations as the heterozygous genotype (HbAS), in which the fractional concentration of HbS is 50%. In our study the mean HbS is 33.3% within a range of 24.5%–41%.^11^, ^12^ The SCD‐related chronic anemia usually induces an increase of the cardiac output and intravascular volume to maintain adequate oxygen delivery. Consequently, left ventricular dilatation, eccentric hypertrophy, and various types of arrhythmias may develop and have been linked to cardiovascular‐related mortality in these patients. While systolic function is typically preserved, diastolic dysfunction frequently occurs, which is recognized as an independent risk factor for mortality in patients with SCD.^13^, ^14^ Chronic hemolytic anemia results in the release of free hemoglobin and other red blood cell intracellular enzymes which inhibits nitric oxide and signals pathways causing vasoconstriction.^13^ Within the pulmonary vasculature, this mechanism is responsible for the development of pulmonary hypertension. Pulmonary hypertension, either secondary to volume overload and underlying diastolic heart failure, or as a primary pathology, is encountered in up to 60% of adult patients with SCD and contributes to early cardiovascular mortality.^13^, ^15^

Unfortunately, some of the patients with SCT developed heart failure in early life, which requires LVAD implantation to improve survival and quality of life for those patients. However, there is a paucity in published evidence on LVAD implantation and its management in SCT patients.

There are many precipitating factors^16^ for sickling including stress, exposure to cold, dehydration, infections, hypoxia, inflammatory cascades, and acidosis. Stress is a major factor that may lead to sickling.^16^, ^17^ Cardiac surgery itself constitutes a major stress for the patient, but the preparatory phase for operation, including intubation and the insertion of catheters, contributes considerably toward this stress and it is strongly recommended that patients must be kept fully sedated during this phase. Such stress conditions lead to potassium efflux, causing formation of insoluble globin polymers. These molecules increase the viscosity of blood and lead to vaso‐occlusive phenomena, which include cell sickling, adherence of sickle cells to the endothelium, and vascular obstruction^11^, ^12^

It should be noted that above‐mentioned predisposing conditions are more common in patients undergoing cardiac surgery. Especially during the operation, CPB itself, as well as aortic cross‐clamping, low‐flow states, topical or whole‐body hypothermia, cold cardioplegia, and use of vasoconstrictive agents, may predispose to the crisis state. Hence, special care should be taken in sickle‐cell patients who require cardiac surgery to avoid or, at least, to minimize those risks factors. These maneuvers may start with decreasing the amount of HbS concentration in the blood with red cell exchange transfusion. We do not use this procedure but the red cell exchange transfusion decreases the amount of circulating sickle cells without increasing hematocrit level or blood viscosity.^18^, ^19^, ^20^ Furthermore, blood transfusion is common during or after any kind of cardiac surgery. Transfusions can be life‐saving for patients with sickle‐cell disease (SCD), but patients may develop antibodies against transfused red blood cells (RBCs) resulting in a delayed hemolytic transfusion reaction (DHTR).^21^ No studies for SCT have been performed. Cell saver was not used, and auto transfusion was not performed during or after surgery.^18^, ^19^ Red blood cells were replenished with packed red blood cells from healthy individuals obtained from the hospital blood bank.^6^, ^12^ Cell‐saver systems are frequently applied during cardiac surgery to conserve blood. Cell‐saver systems include aspiration, a filter wash, and then re‐transfusion of blood to the patient. Intraoperative blood salvage from patients who have sickle cell diseases is an issue that is debated in the medical community. The underlying concern is the possibility that cell salvage blood re‐administered to the patient in question will sickle and further reduce oxygen‐carrying capacity.^20^ There are no trials to support this concern, but, at the same time, the only evidence that supports the administration of cell salvage blood lies in case reports.

Hulatt et al. as described in the safety guidelines the Association of Anaesthetists of Great Britain and Ireland (AAGBI) on the use of intra‐operative cell salvage, advise against the use of cell salvage for those individuals who may require such a blood‐related procedure during their operation. They also indicate that the determination of the re‐administration of cell salvage blood should be examined more on a case to case and individual basis with appropriate and informed consent.^22^ Certainly, further study is required in this area. At the same time, when considering the use of cell salvage, the decision should be made according to risk/benefit determinations on an individual patient basis.^20^ In our series, patients received transfusions in the postoperative period but none presented adverse phenomena. Most likely, this occurred since no use cell‐saver system was adopted.

The potential risk of sickling within the coronary arteries with the administration of cold cardioplegia under the cross‐clamp was avoided with an initial normothermic (36°C) blood cardioplegia dose until cardiac arrest; it was followed by a full blood cardioplegia dose of 20 mL/kg at normothermic temperature. Subsequently, 10 mL/kg blood cardioplegia was administered every 20 min.^23^

The REMATCH trial,^24^ which compared medical therapy to the HeartMate XVE, had a higher rate of adverse events in the LVAD group as described, but every subsequent trial has shown drastic improvements in rates of adverse events. This is especially evident in the trials of MOMENTUM trial which is the device used in the patients in this report. LVAD‐related thrombosis, in contrast to conventional vascular damage or inflammation‐mediated thrombosis, is largely driven by supraphysiologic levels of shear stress imparted to blood elements, notably platelets, and to anemia upon passage through the pump. In our series, we did not find an increase in thrombotic or hemorrhagic events in any patient.

Finally, LVADs have emerged as a mainstay of therapy for patients with advanced refractory left ventricular heart failure (HF). In recent years, a shift from large bulky pulsatile systems to small, rotary continuous flow pumps has been witnessed. With this shift in design, a progressive increase in survival has been observed. However, despite this overall outcome improvement, an accompanying rise in adverse events, notably device‐related thrombosis, thromboembolic events, and adverse neurologic sequelae, has been detected. In our series, we did not observe any cases of thromboembolic events.

## 5 CONCLUSION

Based upon our experience, LVAD implantation in a sickle‐cell trait is safe and offers a remarkable improvement in patient outcomes. In spite of the limited numbers of patients enrolled in this case‐series, the findings suggest that LVAD implantation can be successfully performed in patients with SCD trait by applying meticulous preoperative, intra‐operative, anesthetic, and postoperative management protocols. However, the literature on the specific evaluation and management of these patients remains limited and further studies are still needed to provide more evidence on this practice.
